# Supporting primary care through symptom checking artificial intelligence: a study of patient and physician attitudes in Italian general practice

**DOI:** 10.1186/s12875-023-02143-0

**Published:** 2023-09-04

**Authors:** Angelika Mahlknecht, Adolf Engl, Giuliano Piccoliori, Christian Josef Wiedermann

**Affiliations:** 1 Institute of General Practice and Public Health, College of Health Care Professions (Claudiana), Lorenz Böhler Street 13, 39100 Bolzano, Italy; 2 Department of Public Health, Medical Decision Making and HTA, University of Health Sciences, Medical Informatics and Technology, Eduard-Wallnöfer Place 1, 6060 Hall, Austria

**Keywords:** Feasibility study, COVID-19, General practice, Self-management, Digital technology, Symptom checker, Chatbot

## Abstract

**Background:**

Rapid advancements in artificial intelligence (AI) have led to the adoption of AI-driven symptom checkers in primary care. This study aimed to evaluate both patients' and physicians' attitudes towards these tools in Italian general practice settings, focusing on their perceived utility, user satisfaction, and potential challenges.

**Methods:**

This feasibility study involved ten general practitioners (GPs) and patients visiting GP offices. The patients used a chatbot-based symptom checker before their medical visit and conducted anamnestic screening for COVID-19 and a medical history algorithm concerning the current medical problem. The entered data were forwarded to the GP as medical history aid. After the medical visit, both physicians and patients evaluated their respective symptoms. Additionally, physicians performed a final overall evaluation of the symptom checker after the conclusion of the practice phase.

**Results:**

Most patients did not use symptom checkers. Overall, 49% of patients and 27% of physicians reported being rather or very satisfied with the symptom checker. The most frequent patient-reported reasons for satisfaction were ease of use, precise and comprehensive questions, perceived time-saving potential, and encouragement of self-reflection. Every other patient would consider at-home use of the symptom checker for the first appraisal of health problems to save time, reduce unnecessary visits, and/or as an aid for the physician. Patients’ attitudes towards the symptom checker were not significantly associated with age, sex, or level of education. Most patients (75%) and physicians (84%) indicated that the symptom checker had no effect on the duration of the medical visit. Only a few participants found the use of the symptom checker to be disruptive to the medical visit or its quality.

**Conclusions:**

The findings suggest a positive reception of the symptom checker, albeit with differing focus between patients and physicians. With the potential to be integrated further into primary care, these tools require meticulous clinical guidance to maximize their benefits.

**Trial registration:**

The study was not registered, as it did not include direct medical intervention on human participants.

**Supplementary Information:**

The online version contains supplementary material available at 10.1186/s12875-023-02143-0.

## Background and objectives

The increasingly older-aged population and rising number of patients requiring medical consultations for non-urgent conditions puts intense strain on general practitioners (GPs) with increased demand for appointments and growing workload [1], and the GP workforce is declining [2]. This in turn entails a more difficult access to primary care for patients and leads to lower patient satisfaction with the service [3, 4].

Digitalization may contribute to addressing this issue by enhancing patient self-management and the more effective use of health resources. Digital technologies as an alternative to face-to-face consultations have been endorsed in various healthcare systems [5, 6]; however, it has not yet been sufficiently elaborated under what conditions and for which patients these approaches may offer benefits.

During the COVID-19 pandemic, telephone and video consultations increased considerably [7, 8], while the number of face-to-face visits to hospitals, general practices, and emergency departments decreased significantly [7, 9, 10]. Although patients were also unable to access indispensable care, this phenomenon suggests that some of the reduced treatments may have been unnecessary, implying the risk of iatrogenic harm. Thus, the pandemic has opened the opportunity to intensify efforts to reduce unnecessary care, which in turn could prevent avoidable patient harm and improve the sustainability of healthcare [9].

Symptom checkers with chatbots are becoming increasingly common in general practice, as they offer patients a convenient and accessible way to check their symptoms and seek advice. These applications are based on chatbots, that is, computer programs that can perform conversations with users using Artificial Intelligence (AI) algorithms [11]. A potential benefit is that they can help reduce GP workloads by providing patients with a self-service option for the initial symptom assessment. In this way, GPs can focus on more complex cases and provide personalized care to patients who need it.

Symptom checkers provide a list of potential differential diagnoses based on the entered symptoms and other parameters [12] and are usually ranked according to their likelihood [13]. A further function of several symptom checkers is triage advice that suggests a course of action (e.g., self-care, seeking the GP or an emergency department) and its level of urgency [13]. Symptom checkers may support the management and remote care of COVID-19 [14] and other common health problems.

However, to date, it has not been sufficiently demonstrated whether and how symptom checkers can beneficially support general practice [15]. Previous studies concluded that symptom checkers had deficits in both triage and diagnosis and that triage advice was generally risk-averse, encouraging users to seek care for conditions where self-care was reasonable [13, 16]. However, symptom checkers receive attention from governments and the scientific community as a way in which individuals could be encouraged to self-manage, and emergency and primary care services could subsequently be disburdened.

Patient and physician attitudes towards using a chatbot for symptom checking can affect their willingness to use the technology. Some patients may feel uncomfortable with the idea of using a chatbot instead of talking to a human doctor, whereas others may welcome the convenience and accessibility of a chatbot. Patients with negative attitudes towards technology may also be less likely to use a chatbot for symptom checking.

For the present study conducted during the pandemic, an existing symptom checker combining COVID-19 and non-COVID-19 medical problems was piloted in northern Italian GP offices. Patients visiting the participating GP offices used the symptom checker before the medical visit by first responding to COVID-19-related questions and then answering questions related to the current health problem. The aim of this study was to determine whether symptom checking in general practice is viable and feasible in the Italian healthcare system and to identify potential risks and challenges that could affect its success.

In the context of primary care, particularly in Italian settings, there is a paucity of research evaluating the perspectives of both physicians and patients regarding the use of these symptom checkers. While physicians may evaluate these tools based on their clinical utility, patients may have a different set of evaluation criteria, focusing on aspects such as empowerment, ease of use, and clarity of information.

The following research questions were investigated:

1. How do patients perceive the use of AI-driven symptom checkers in terms of empowerment, user friendliness, and overall satisfaction?
2. What are the perceived benefits and challenges of these tools, as identified by physicians, especially concerning differential diagnosis?
3. Can these AI-driven symptom checkers be integrated seamlessly into the regular workflows of general practices and, if so, how?

With these research questions in mind, this study aims to bridge the knowledge gap by offering insights into the attitudes of both physicians and patients towards AI-driven symptom checkers in Italian general practice settings.

## Methods

### Study design and population

The study was conducted in northern Italy (Province of Bolzano) and involved GPs and adult patients seeking treatment at the GP office. Owing to the feasibility of the study design, no power calculations were performed. The aim was to recruit 10 GP offices and up to 100 patients per GP office (up to 1000 patients in total) to obtain meaningful results.

The practice phase started on 2021/09/27 and was scheduled to end on 2021/11/26. The GPs were given the option to further extend the practice phase; four GPs agreed and concluded the study on 2021/12/10.

### Inclusion and exclusion criteria for the study participants

*GPs*: Experience of scientific work or former study participation, interest in digitalization and quality improvement in primary care, presence of a medical assistant in the GP office for supporting the study procedures, and informed consent to participate were the inclusion criteria for GPs.

*Patients*: Adults with any newly occurring medical problem or symptom, direct contact with the GP (medical visit), ability to use a tablet-PC, sufficient German or Italian language skills (the two main languages spoken in the Province of Bolzano), ability to read and understand the study information and chatbot text, and informed consent to participate were included in the study.

Pregnancy, patient not visiting the GP office (e.g. home visits), patients visiting the GP office for other reasons than medical problems (e.g. merely administrative contacts), severe symptoms or emergencies requiring immediate medical actions, and markedly impaired health status were exclusion criteria for patients; furthermore, patients were excluded from the study after the first part of the symptom checking procedure in case of a medical history medium or high risk for COVID-19 as estimated by the symptom checker (see below).

### Recruitment and information of study participants

#### GP recruitment

All GPs listed in the Chamber of Physicians of Bolzano were invited to participate in the study through an official announcement on the website of the Institute of General Practice and Public Health (IGP) in Bolzano, Italy. We additionally applied convenience sampling; GPs with known experience in study participation were contacted by email and/or phone and received a detailed information letter.

Ten GPs willing to participate and fulfilling the inclusion criteria were enrolled and signed informed consent forms were obtained. The participating physicians were involved in the development of detailed study procedures in the GP offices to ensure practicality for GPs, medical assistants, and patients.

Before the beginning of the study, all participating GPs were instructed in two video meeting sessions (2021/09/23) and received written instructions on how to use the symptom checker and the study dashboard.

To acknowledge the efforts of the participating GPs, remuneration based on the number of patients included in the study was granted.

#### Patient recruitment

In the participating GP offices, handouts (fly sheets) were available to inform patients about the study. Consecutive patients visiting the GP office and fulfilling the inclusion criteria were identified by the GPs or by medical assistants under the supervision of the GPs and were invited to participate prior to the medical visit.

Patients interested in participation were explained the study procedures through a detailed information section, which was displayed digitally when opening the symptom checker on the tablet. The patient information section contained all patient-relevant study information in a generally intelligible language.

Informed consent for the patients was included digitally in the symptom checker after the information section and signed online by explicitly agreeing to participate.

All study materials for patients and GPs (information letter, consent form, symptom checker, and questionnaires) were delivered in German and Italian.

The study was presented on a local television channel in early November 2021 to publicly advertise the general population and to increase patient participation.

**Fig. 1:**
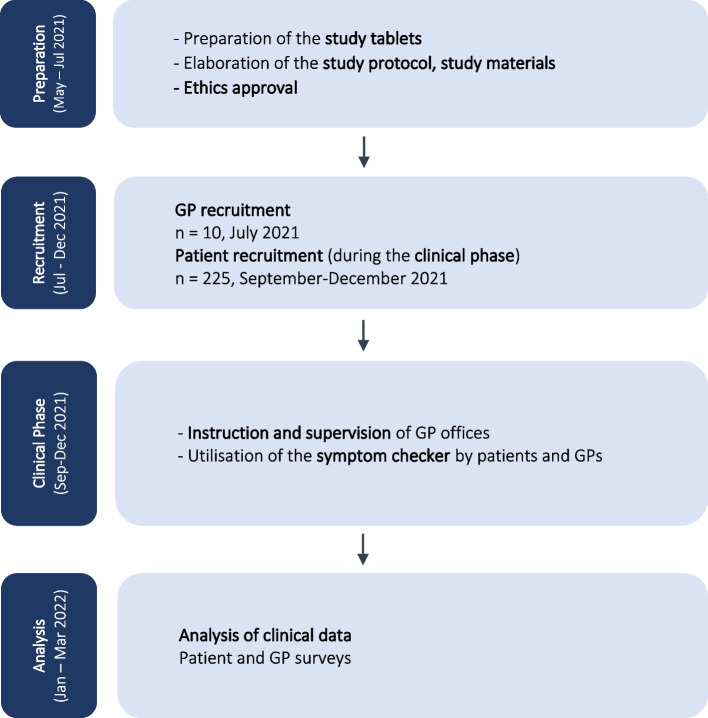
Study flowchart: Study phases and timeline. *GPs* General practitioners

Figure 1 shows an overview of the study procedures and responsibilities of the partners.

### Symptom checker with chatbot

Symptom checkers are offered online, free of charge but also partly for a fee. As the focus of this study is on the feasibility of its use in general medical consultations and not on a comparative evaluation of the different ways in which artificial intelligence is used, the symptom checker used in this study will not be identified. The symptom checker used was validated by physicians and was based on a medical database that connects symptoms and conditions. It provides information on the possible causes of the symptoms that the user enters. In this study, a digital health assistant was used in German and Italian.

The output of the symptom checker, which was generated based on the information entered by the patient, listed potential differential diagnoses ranked according to their likelihood, without any triage advice. This output was *not* used as a diagnosis or treatment recommendation, but only indicated that persons with similar symptoms and risk factors might have a specific condition based on AI and the scientific literature data used. The GPs were instructed to critically appraise the chatbot overview and not use it to replace or distort their own anamnesis and diagnosis. The decision regarding medical actions always belonged to the GP, together with the patient, in an informed decision-making process.

### Practice phase

Every participating GP office was provided with two tablet computers on which the symptom checker was accessible. Patients who were willing to participate were given a tablet in the waiting room. As the tablets were successively used by different patients, hygienic rules and strategies for the prevention of infection were thoroughly followed.

When the patient opened the symptom checker app, a detailed information section appeared. At the end of this section, patients were asked about their consent or refusal to participate in the study. By clicking ‘not agree,’ the symptom checker questions did automatically not proceed.

After electronically providing informed consent, the patients filled in the symptom checker on the tablet during the time they spent waiting for the medical visit. The medical staff provided technical assistance when needed. Using a symptom checker at home or by means of individual devices was explicitly not part of the study. The patients intended to participate only once in the study.

The symptom checking procedure consisted of two consecutive parts:

- *Part 1 – Anamnestic screening for COVID-19*: The symptom checker asked the patient about potential COVID-19-related symptoms. If the symptom check resulted in a ‘low risk’ of COVID-19, the second part of the symptom check proceeded. Inclusion in the full study procedure was only possible for patients classified as ‘low risk’ of COVID-19 by the symptom checker.
  In case of an estimated ‘medium’ or ‘high risk’ of COVID-19, the tool ended the symptom checking procedure and instructed the patient to approach the medical assistant. The medical assistant evaluated and coordinated further actions with the GP (e.g., considering diagnostic testing for COVID-19 and proper isolation of the patient from other patients).
- *Part 2: Symptom checking based on today’s medical problem* that induced the patient to seek the GP.

After the patient had finished the entries in the symptom checker, the entered information was forwarded electronically to the GP, who accessed the patient’s information on their own computer. For this purpose, a secure online study dashboard was created on which patient information was displayed using a digital anamnestic sheet. The GPs used this anamnestic sheet before and during their personal contact with the patient. For each new patient who used the symptom checker, the GP received a notification on the dashboard. Every GP was only able to access the patient’s data.

**Fig. 2:**
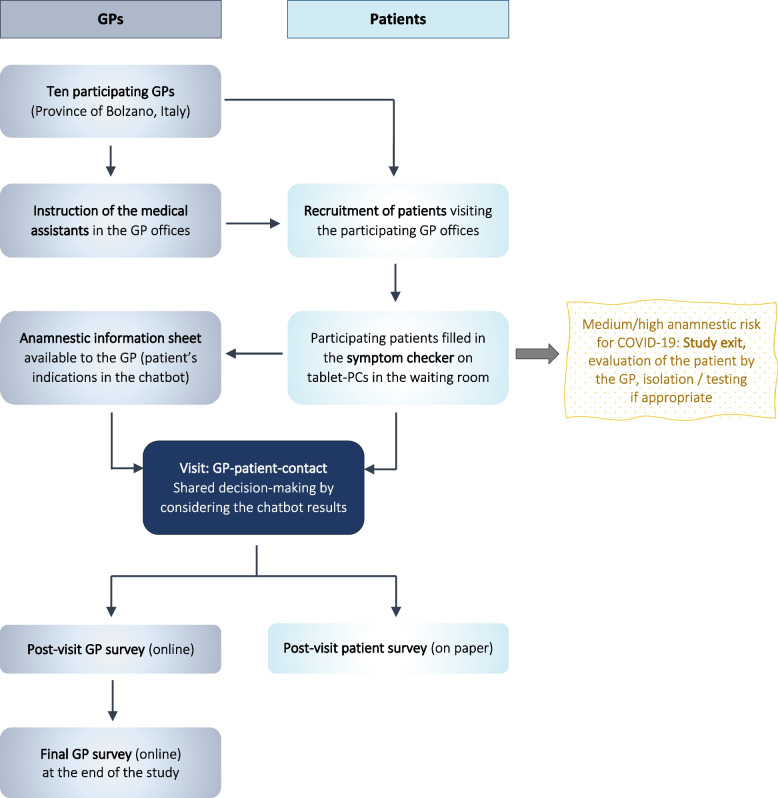
Clinical study phase: Implementation and evaluation of the symptom checker in the GP offices. *GPs* General practitioners

The study team supervised the general practice team regarding procedures and technical issues. Supervision was provided online and via telephone. Figure 2 presents an overview of the study procedures in the GP offices.

### Evaluation of the symptom checker

#### Post-visit evaluation of the symptom checker

After the medical visit, both GPs and patients were surveyed ad hoc through a short quantitative evaluation of their experiences with the symptom checker. Two different questionnaires for GPs and patients were developed for this purpose. The GP accessed the pseudonymized questionnaire online on the study dashboard; the patients received an anonymous paper questionnaire that they returned to the medical assistant upon completion.

The questionnaires covered the following aspects:

- Demographic characteristics, health status, purpose of the consultation, and result of the GP visit (discharge in self-management, re-contact with the GP, prescription of drugs, referral to a diagnostic procedure/specialist visit, admission to hospital or emergency department).
- Experience and satisfaction of physicians and patients regarding digital symptom checking, influence on the content and/or the quality of the consultation, impact on the time needed for the medical visit, if it was helpful or disturbing for the GPs to receive chatbot-generated data, and concordance between the symptom checker result and the diagnosis or medical appraisal of the GP.

#### Final evaluation of the symptom checker

At the end of the study, GPs were further surveyed to explore their overall experiences with the symptom checker. The final GP questionnaire was delivered online using the survey tool ‘Q-set’ (www.q-set.de).

### Collection of data

Data were collected, exported, and analyzed using the pseudonymization ID codex. Two main categories of data were collected: (i) data derived from the questionnaire results and (ii) data collected during the use of the symptom checker. This article reports questionnaire-related data.

### Questionnaire-related data

Each post-visit questionnaire was given a pseudonymization ID corresponding to the numeric symptom checker ID of the respective patient. No identification or visualization of the patient names was possible for the research team at any time.

The GPs were assigned a number that was used for the pseudonymization and analysis of the final questionnaires.

Responses to the online questionnaires were automatically transferred into a CSV or Excel®-datafile, which was subsequently exported for analysis. Data from the paper-based questionnaires were entered manually by the research team.

### Patient data collected during the symptom checking sessions

- Age and sex (optional, helped the symptom checker to find possible causes more precisely).
- Sensitive (health-related) data were collected in a completely anonymous manner, that is, all users were rendered completely unrecognizable by assigning a pseudonymization ID.

Only the GPs were able to access the personalised data of their specific patients.

### Data analysis

Statistical analyses were conducted using IBM®SPSS®Statistics version 25.0. Descriptive statistics, including absolute/relative frequencies, medians/interquartile range (IQR), and cross-tabulations, were calculated. Free-text comments were categorized and summarized descriptively. Spearman correlations and chi-square tests were used for the subgroup analyses (Supplementary Table II). All tests were two sided. The level of statistical significance was set at *p* < 0.05. Only completed questionnaires were considered for analysis. In case of single missing responses, the concerned individuals were excluded from the analysis of the respective items.

For participant demographic segmentation, we categorized individuals aged 50 and above as the "older" cohort. This age threshold is significant, as it captures the transition between the Baby Boomer and Generation-X cohorts. Baby Boomers, many of whom crossed the age of 50 in our study period, had to adapt to digital technologies in their adult lives. Generation X members are generally more familiar with technology.

## Results

### Study participants

Ten GPs and 225 patients participated in the study. Of 225 patients, 145 completed the post-visit survey. Of these, 29 patients were post hoc excluded from the analysis due to a chatbot-estimated medium or high anamnestic risk for COVID-19 (according to the study protocol, these patients should have been excluded from the study immediately after the completion of the COVID-19-related symptom check and should not have received a post-visit questionnaire; however, in some cases, this was not correctly observed by the GP offices and the concerned cases were therefore post hoc excluded from the analysis). Thus, 116 post-visit questionnaires (51.6% of 225 participating patients) were included in the final analysis.

Ten GPs completed 127 post-visit questionnaires. Of these, five were post hoc excluded because of patients’ medium/high anamnestic risk for COVID-19, resulting in 122 analyzed GP post-visit questionnaires (i.e., 54.2% of the 225 participating patients).

The median duration of the GPs’ professional experience was 16.5 years, and rural and urban GP offices were equally represented. Eight of the ten participating GPs worked in a group office.

The median age was 54 years (GPs) and 47 years (patients). Half of the GPs and 55.2% of the patients were female.

The patients' most frequently reported educational level was vocational school (39.1%), followed by high school (27.8%). The vast majority of patients (87.1%) indicated that their GP was the primary source of health-related information. Internet-based sources were used by 13.8% of patients.

The patients’ health status was rated as good by most of the patients themselves (61.2%) and very good by the majority of the GPs (44.3%, Table 2). The most prevalent patient-reported chronic conditions were allergies, followed by arterial hypertension and psychological disorders. The leading reasons for seeking GP (today’s medical problem) were pain in the lower limbs (GP-reported frequency:15.6%, patient-reported frequency:15.5%) and asthenia/fatigue (GP-reported,9.8%; patient-reported,4.5%). The most frequent recommendations issued by the GPs during medical visits were drug prescriptions (54.7%). Referrals to diagnostic procedures or specialist visits were prescribed to approximately one-quarter of the patients, mostly at a non-urgent level. The characteristics of the study participants are shown in Tables 1 and 2.

**Table 1:** Demographic characteristics of the participating GPs and patients

Participating GPs (*n* = 10)	n (%)	Median (IQR)
**Gender**	***n* = 10**	
Female	5 (50.0%)	
**Age**		***n* = 10**
		54.0 (45.5 – 57.5)
**Number of assisted patients per GP office**		***n* = 10**
		1685 (1583 – 1739)
**Duration of professional activity [years]**		***n* = 10**
		16.5 (10.0 – 24.5)
**Location of GP office**	***n* = 10**	
Rural area	5 (50.0%)	
Urban area	5 (50.0%)	
**Practice organisation**	***n* = 10**	
Group office	8 (80.0%)	
Network of GP offices	1 (10.0%)	
Single-handed office	1 (10.0%)	
**Participating patients (*n* = 116) **^**i**^	**n (%)**	**Median (IQR)**
**Gender**	***n* = 116**	
Female	64 (55.2%)	
**Age**		***n* = 113**
		47.0 (34.0 – 56.0)
**Educational level**	***n* = 115**	
Basic education	2 (1.7%)	
Intermediate school	9 (7.8%)	
Vocational college	45 (39.1%)	
High school	32 (27.8%)	
University	27 (23.5%)	
**Current working situation**	***n* = 111**	
Employee	62 (55.9%)	
Freelancer	19 (17.1%)	
Retired	16 (14.4%)	
Student	8 (7.2%)	
Maternal leave / housewife	4 (3.6%)	
Professional education	1 (0.9%)	
Job seeking	1 (0.9%)	
**Where do the patients primarily seek health-related information**	***n* = 116**	
Their GP	101 (87.1%)	
Internet: Google, YouTube, social media	16 (13.8%)	
Family members or friends	13 (11.2%)	
Homepages of medical associations and societies	10 (8.6%)	
Pharmacist	8 (6.9%)	
Another physician	4 (3.4%)	
Print media (books, journals)	3 (2.6%)	
Others	1 (0.9%)	

*GP(s)* General practitioner(s), *IQR* Interquartile range

^i^ Demographic information was available only for patients who completed the post-visit survey

**Table 2:** Patients’ health status, reasons for consulting the GP, and results of medical visits

Patients’ health-related information	Appraisal by the GP	Patient-reported
**Health status**	***n* = 122** ^i^	***n* = 116**
Very good	54 (44.3%)	23 (19.8%)
Good	48 (39.3%)	71 (61.2%)
Mediocre	19 (15.6%)	21 (18.1%)
Poor	1 (0.8%)	1 (0.9%)
Very poor	0 (0.0%)	0 (0.0%)
**Chronic conditions**	-	***n* = 105**
Allergies	-	18 (17.1%)
Arterial hypertension	-	16 (15.2%)
Psychologic disorders	-	7 (6.7%)
Metabolic conditions	-	5 (4.8%)
Cardiovascular conditions	-	4 (3.8%)
Pulmonal conditions	-	3 (2.9%)
Immunologic conditions	-	2 (1.9%)
Oncologic conditions	-	1 (1.0%)
Others	-	11 (10.5%)
**Most frequent current reason(s) for seeking the GP**	***n* = 122** ^i^	***n* = 110**
Pain lower extremities	19 (15.6%)	17 (15.5%)
Asthenia, fatigue, exhaustion	12 (9.8%)	5 (4.5%)
Consulting, prescriptions, discussion of results of diagnostic procedures	11 (9.0%)	19 (17.3%)
Abdominal pain, diarrhoea, obstipation	10 (8.2%)	10 (9.1%)
Dermatologic problems	9 (7.4%)	7 (6.4%)
Pain upper extremities	7 (5.7%)	6 (5.5%)
Upper respiratory tract infections, tonsillitis, sore throat	6 (4.9%)	5 (4.5%)
Psychologic disturbances, nervosity, anxiety	5 (4.1%)	3 (2.7%)
Pain without specification	5 (4.1%)	2 (1.8%)
Arterial hypertension	4 (3.3%)	5 (4.5%)
Otalgia, otitis	4 (3.3%)	3 (2.7%)
Pruritus	4 (3.3%)	2 (1.8%)
Low back pain	3 (2.5%)	5 (4.5%)
Hypoacusis, ear noise	3 (2.5%)	4 (3.6%)
Dyspnoea, unspecified respiratory problems, snoring	3 (2.5%)	3 (2.7%)
Chest pain	3 (2.5%)	3 (2.7%)
Micturition disorders	3 (2.5%)	2 (1.8%)
Increased perspiration	3 (2.5%)	not indicated
Epigastric pain, heartburn, reflux	2 (1.6%)	6 (5.5%)
Eye problems	2 (1.6%)	2 (1.8%)
Sleeping disturbances	2 (1.6%)	2 (1.8%)
Varices / haemorrhoids	2 (1.6%)	2 (1.8%)
Hair loss	2 (1.6%)	2 (1.8%)
Cough	2 (1.6%)	1 (0.9%)
Epileptic seizures	2 (1.6%)	1 (0.9%)
Cervicalgia	2 (1.6%)	not indicated
Allergy, chronic rhinitis	2 (1.6%)	not indicated
Vertigo	1 (0.8%)	4 (3.6%)
Post-COVID-19 symptoms	1 (0.8%)	3 (2.7%)
Headache	1 (0.8%)	2 (1.8%)
**Recommendations and/or prescriptions issued by the GP**	-	***n* = 106**
Prescription of medical therapy	-	58 (54.7%)
Prescription of / referral to diagnostic measures	-	26 (24.5%)
Referral to specialist visit	-	25 (23.6%)
Discharge at home in self-observation	-	14 (13.2%)
Physical re-contact with the GP (medical visit)	-	10 (9.4%)
Telephonic re-contact with the GP	-	9 (8.5%)
Referral to emergency department	-	0 (0.0%)
**Level of urgency of prescribed diagnostic measures / specialist visits**		***n* = 39**
Within 24 h	-	2 (5.1%)
Within 10 days	-	8 (20.5%)
Within ≥ 30 days	-	29 (74.4%)

*GP(s)* General practitioner(s)

^i^ total of 122 post-visit questionnaires were completed by participating GPs throughout the study period

### Post-visit evaluation of the symptom checker by patients and GPs

The numeric questions regarding experiences with the symptom checker showed only occasional missing values, while free-text indications were given by fewer participants (up to 12.7% of the patients participating in the post-visit survey and 22.1% of the GPs). The complete numerical results are shown in Table 3, and the free-text indications are depicted in detail in Supplementary Table I a.

**Table 3:** Patients’ and GPs’ experiences with the symptom checker (post-visit questionnaires): quantitative results

General satisfaction with the symptom checker		
	**Patients (*n* = 110)**	**GPs (*n* = 122) **^**i**^
Very satisfied	21 (19.1%)	21 (17.2%)
Rather satisfied	33 (30.0%)	12 (9.8%)
Neutral	51 (46.4%)	60 (49.2%)
Rather dissatisfied	3 (2.7%)	19 (15.6%)
Very dissatisfied	2 (1.8%)	10 (8.2%)
**Quality of the chatbot result from a clinical point of view**		
	**-**	**GPs (*n* = 122)**
Very appropriate	-	22 (18.0%)
Rather appropriate	-	16 (13.1%)
Neutral	-	54 (44.3%)
Rather inappropriate	-	19 (15.6%)
Very inappropriate	-	11 (9.0%)
**Concordance of the chatbot result with the GP’s clinical appraisal**		
	**-**	**GPs (*n* = 122)**
Completely concordant	-	27 (22.1%)
Rather concordant	-	35 (28.7%)
Rather not concordant	-	41 (33.6%)
Not concordant at all	-	19 (15.6%)
**Impact of the use of the symptom checker on the quality of the medical visit**		
	**Patients (*n* = 111)**	**GPs (*n* = 122)**
Very positively	6 (5.4%)	8 (6.6%)
Rather positively	23 (20.7%)	18 (14.8%)
Neutral	81 (73.0%)	93 (76.2%)
Rather negatively	1 (0.9%)	2 (1.6%)
Very negatively	0 (0.0%)	1 (0.8%)
**Helpfulness of the symptom checker for the medical visit**		
	**Patients (*n* = 110)**	**GPs (*n* = 122)**
Very helpful	5 (4.5%)	9 (7.4%)
Rather helpful	23 (20.9%)	15 (12.3%)
Neutral	62 (56.4%)	75 (61.5%)
Rather not helpful	8 (7.3%)	9 (7.4%)
Not helpful at all	12 (10.9%)	14 (11.5%)
**Disturbance of the medical visit by the use of the symptom checker**		
	**Patients (*n* = 110)**	**GPs (*n* = 122)**
Very disturbing	0 (0.0%)	1 (0.8%)
Rather disturbing	3 (2.7%)	0 (0.0%)
Neutral	33 (30.0%)	59 (48.4%)
Rather not disturbing	17 (15.5%)	15 (12.3%)
Not disturbing at all	57 (51.8%)	47 (38.5%)
**Were the patients’ indications on the symptom checker considered by the GP?**		
	**Patients (*n* = 102)**	**-**
Yes	80 (78.4%)	-
No	22 (21.6%)	-
**Duration of the medical visit compared with the time expected**		
	**Patients (*n* = 113)**	**GPs (*n* = 122)**
Much shorter	3 (2.7%)	1 (0.8%)
Rather shorter	10 (8.8%)	7 (5.7%)
Unvaried	85 (75.2%)	102 (83.6%)
Rather longer	15 (13.3%)	11 (9.0%)
Much longer	0 (0.0%)	1 (0.8%)
**Subjective valuation of the duration of the medical visit**		
	**Patients (*n* = 115)**	**GPs (*n* = 122)**
Too short	2 (1.7%)	1 (0.8%)
Adequate	109 (94.8%)	115 (94.3%)
Too long	4 (3.5%)	6 (4.9%)
**Future at-home use of the symptom checker as an aid to the appraisal of health problems**		
	**Patients (*n* = 115)**	**-**
Yes, surely	28 (24.3%)	-
Rather yes	26 (22.6%)	-
Neutral	27 (23.5%)	-
Rather not	28 (24.3%)	-
Absolutely not	6 (5.2%)	-
**Satisfaction with the usability of the symptom checker**		
	**Patients (*n* = 115)**	**-**
Very satisfied	27 (23.5%)	-
Rather satisfied	48 (41.7%)	-
Neutral	28 (24.3%)	-
Rather dissatisfied	8 (7.0%)	-
Very dissatisfied	4 (3.5%)	-

*GPs* General practitioners, *DDs* Differential diagnoses

^i^ total of 122 questionnaires were completed by participating GPs throughout the study period

Most patients (87.8%) had never used a symptom checker previously. Overall, 49.1% of patients and 27.0% of GPs were satisfied or very satisfied with the symptom checker. The most frequent patient-reported reasons for *general satisfaction* (free-text indications) were easy and rapid use of the symptom checker, precise and comprehensive questions, perceived time-saving potential, and encouragement of self-reflection. Dissatisfaction was less frequently expressed and concerned impersonality, generic questions, and the complex use of digital tools.

The most frequent GP-reported reason for patient satisfaction was the output of adequate differential diagnoses and helpful indications. GPs were dissatisfied in some cases because of inadequate differential diagnoses and the inability of patients to correctly enter symptoms in the symptom checker.

The *clinical quality of the chatbot result* was rated as rather or very appropriate by 31.1% of the GPs and as rather or very inappropriate by 24.6% of the GPs, while half of the GPs considered the result of the symptom checker concordant, which was not concordant with their clinical appraisal.

Approximately one-quarter of the patients and one-fifth of the GPs reported a rather or very positive *impact on the quality of the medical visit* while a negative impact was only occasionally observed. The GPs mainly attributed the positive impact to the support provided by the alternative diagnoses and to positive effects on the patients who had used the symptom checker (increased preparation for the medical visit, enhanced attentiveness, positive reinforcement). The patients reported that conveying information to the GP before the start of the visit had the most positive impact on medical visits.

One-quarter of the patients and nearly one-fifth of the GPs rated the symptom checker as rather helpful or very *helpful for the medical visit*. However, about one-fifth of both GPs and patients considered the tool unhelpful. The most frequently mentioned helpful aspects were a confirmation of the suspected diagnosis, a supporting list of alternative diagnoses (GPs), and the pre-visit information for the GP (patients).

Issues mentioned by the patients as unhelpful were the initial focus of the symptom checker on COVID-19-related symptoms rather than on the current medical problem. The GPs reported inadequate differential diagnoses in some cases as unhelpful for the visit.

A *disturbing effect on the medical visit* was mentioned by a few study participants (2.7% of the patients, 0.8% of the GPs).

Most patients (75.2%) and GPs (83.6%) had no impact of the symptom checker on the *duration of the visit*. A shortening effect was observed in 11.5% of the patients and 6.5% of the GPs; however, 13.3% of the patients and 9.8% of the GPs perceived a prolongation of their medical visits. More than 90% of both patients and GPs considered the duration of the medical visit as adequate; however, 3.5% of patients and 4.9% of GPs perceived the duration as too long.

About 47% of the patients would consider a *future at-home use of the symptom checker* for the first appraisal of health problems, mainly to save time, to reduce unnecessary visits, and/or as an aid for the GP. On the other hand, 29.5% of the patients indicated not using the symptom checker at home, mostly due to a preference for personal contact with the GP or because they saw no additional benefit of a symptom checker.

The *usability of the symptom checker* was rated as satisfactory by nearly two-thirds of the patients (65.2% were rather satisfied or very satisfied).

Patients’ attitudes towards the symptom checker were not significantly associated with age, sex, or level of education (Supplementary Table II). Patients aged 50 + and those with a lower level of education reported a non-significantly higher general satisfaction and a more helpful impact of the symptom checker on medical consultation. Male patients and persons aged 50 + years showed a non-significantly more positive attitude towards future at-home use of the symptom checker.

### Final evaluation of the symptom checker by the GPs

The numerical results of the final evaluation are summarized in Table 4, and the free-text indications are listed in Supplementary Table I b.

**Table 4:** GPs’ experiences and evaluation of the symptom checker (final survey, *n* = 10 GPs): Quantitative results

**GPs’ general satisfaction with the symptom checker**	
Very satisfied	0 (0.0%)
Rather satisfied	1 (10.0%)
Neutral	3 (30.0%)
Rather dissatisfied	5 (50.0%)
Very dissatisfied	1 (10.0%)
**Do GPs consider the use of the symptom checker as helpful for patients’ self-management?**	
Very helpful	1 (10.0%)
Helpful	2 (20.0%)
Neutral	3 (30.0%)
Rather not helpful	4 (40.0%)
Not helpful at all	0 (0.0%)
**Probability that GPs recommend the use of the chatbot before or as alternative to a medical visit**	
Yes, surely	0 (0.0%)
Rather yes	1 (10.0%)
Neutral	4 (40.0%)
Rather not	2 (20.0%)
Not at all	3 (30.0%)
**Do GPs consider the symptom checker useful to reduce unnecessary visits?**	
Very helpful	0 (0.0%)
Helpful	3 (30.0%)
Neutral	0 (0.0%)
Rather not helpful	4 (40.0%)
Not helpful at all	3 (30.0%)
**Do GPs consider specific patient groups as especially suited for the use of the symptom checker?**	
Yes	7 (70.0%)
No	3 (30.0%)

*GPs* General practitioners

The final *general satisfaction* was rated as rather high by one GP (10.0% of the ten participating GPs) and rather/very low by six GPs (60.0%). Dissatisfaction (free-text answers) was expressed regarding the temporal difficulties of the study implementation due to the high pandemic-related workload, frequent dropout of patients due to exclusion criteria, logistical inconveniences for the medical staff and patients, technical issues, and the low perceived usefulness of the alternative diagnoses provided by the tool. In contrast, two GPs (20.0%) were reported to be satisfied because of the usefulness of the provided differential diagnoses.

Three GPs (30.0%) considered the symptom checker useful for *future patient self-management*, mostly for the differentiation and recognition of hazardous situations. Non-usefulness for self-management was mainly attributed to concerns of complexity and technical issues, lack of clinical examination, and preference for direct contact with the patient.

Half of the GPs would not recommend the *use of the symptom checker before or as an alternative to a medical visit*, and four GPs (40.0%) were uncertain in this regard. The reasons for not being likely to recommend the symptom checker were the indispensability of the GP’s judgement and clinical examination, the perceived difficulties of the patients in autonomously handling the symptom checker and explaining their symptoms, and the lack of suitability of the symptom checker for specific (e.g., anxious) patients. One GP (10.0%) would recommend a symptom checker in cases of doubt or for considering rare conditions as alternative diagnoses.

For a potential *future reduction of unnecessary medical visits*, three GPs (30.0%) considered the symptom checker helpful, while seven GPs (70.0%) valued the tool as not helpful in this regard.

The majority of the GPs (70.0%) considered the chatbot suitable for *specific patient groups*, mainly for young patients with experience in the utilization of digital devices and patients with benign and non-complex conditions.

In general, four GPs (40.0%) mentioned that high pandemic-related workload was the most impeding issue in the implementation of the study. However, the approach was rated as interesting by three GPs (30.0%).

## Discussion

The use of symptom checkers in general practice could contribute to increasing the efficiency of healthcare use in primary care against the background of an age-related increase in workload and a simultaneous decrease in the number of GPs practicing. Before introducing the broader use of this digital health tool with artificial intelligence in everyday clinical practice in various healthcare systems, the efficacy, safety, and cost-effectiveness must be tested in an evidence-based manner. For the design of corresponding studies, it is necessary that attitudes towards the use of symptom checkers are known to both patients and GPs. This study assessed the experiences of patients and physicians who used a symptom checker with a chatbot within their daily routines in the primary care setting.

Not surprisingly, in the present study, patients' attitudes towards the use of symptom checkers in primary care settings were more positive than those of the GPs. Patients may feel empowered and under control of their health when using symptom checkers, whereas some GPs may view symptom checkers as a potential threat to their professional expertise. Patients were more accepting of the limitations of symptom checkers, whereas GPs were more skeptical of their accuracy and usefulness in clinical practice. Neither patients nor GPs saw symptom checkers as a way to save time for medical consultation. Patients may be more willing to trust the information provided by symptom checkers, whereas GPs are more critical of the quality and reliability of algorithms used to generate diagnostic recommendations. As the majority of patients in our study did not have previous experiences with symptom checkers, the attitudes observed are unlikely to reflect impressions from the past.

Regarding *general satisfaction*, two main aspects were highlighted: (i) the patients’ satisfaction, although modest, was considerably higher than the satisfaction expressed by the GPs and (ii) the levels of the GPs’ satisfaction notably diverged between the post-visit evaluations, which were given immediately after the respective consultation, and the final survey, which took place after the conclusion of the practice phase. This especially applies to the usefulness of differential diagnoses that was rated higher throughout the post-visit survey.

For the patients, the study approach held a potential of interesting newness while being related to few efforts, as they could use the symptom checker in the waiting room and had no additional tasks to perform (except the completion of the post-visit questionnaire, which was, however, optional). However, some patients mentioned that they preferred personal contact with the physician.

For the GPs, the study added some working steps, which were particularly challenging to integrate into the daily workflow in autumn 2021 when the pandemic-related workload grew even more intense than in previous periods [17]. Rising respiratory infections in this period contributed to more frequent chatbot estimations of medium/high risk for COVID-19, and thus, to lower numbers of eligible patients than a priori expected. This might have additionally reduced the final GPs’ satisfaction because they were remunerated according to the number of included patients. Thus, the ratings of the GPs are probably affected by external circumstances and cannot represent a fully reliable content-related evaluation.

Other studies examining the impact of symptom checkers showed higher satisfaction rates among patients; however, these studies only addressed COVID-19-related symptom checkers [14] or were conducted before the onset of the COVID-19 pandemic and did not include critical appraisal by physicians. A recent RCT showed a high acceptance of conversational chatbot interventions among patients with chronic pain [18]. Although no alteration of pain-related impairment (main outcome) was achieved in this study, the high user satisfaction may suggest that chatbots in the future might be beneficially applied not only by generally healthy persons, but also by those with chronic conditions or presenting with additional unclear symptoms. This could be particularly interesting in general practice, where chronic care plays a major role. Although a proof of safety and of positive impacts on clinical outcomes by digital health tools could be considered a precondition for implementing their broader use, future studies in the primary care setting should also investigate whether their use by patients is able to beneficially influence the patients’ interaction with the GP and the physician–patient relationship [19].

The *clinical quality of the chatbot result* was inconsistently rated by the GPs, and only half of the GPs valued the chatbot results as concordant with their own clinical appraisal. These results suggest that there is still room for improvement in the accuracy of symptom checkers in general practice. Nevertheless, a remarkable part of the GPs considered the provided alternative diagnoses as adequate or interesting, while inappropriate diagnoses were less frequently mentioned (Supplementary Table I a). Thus, the potential of symptom checkers regarding their usefulness for GPs can be noted, which could be increased by further development and adaptation to the primary care setting.

From the feedback of the GPs in the present study, hardly any conclusions can be drawn regarding the diagnostic accuracy of the symptom checker. In our study, the use of the symptom checker was embedded in a real-life setting and the chatbot results were subjectively valued by physicians. A feasibility study envisaged an initial COVID-19 triage during the pandemic and, subsequently, the general medical work-up of an emerging health problem was performed, thus precluding unbiased data sampling.

A recent study measuring the diagnostic accuracy and triage advice of various symptom checkers against standardized clinical case vignettes found that the formerly detected weaknesses [13] in diagnosis and triage did not substantially improve [20]. Although the assessed tools tended to be slightly less risk-adverse than in former analyses [13], they still did not adequately perform regarding advice of self-management, which would be the most important feature for disburdening healthcare providers [20, 21]. A recent systematic review [22] confirmed a low overall diagnostic accuracy (18%-48% for primary care conditions), while the triage accuracy was higher (49%-90%). As both under-triaging and over-triaging may entail deleterious effects (non-detection of potentially hazardous situations or overuse of healthcare services) and variability between symptom checkers entails safety concerns [22], transparent research and guideline development should be promoted [22, 23]. However, a previous systematic review found no evidence of detrimental impact on patient safety [16].

In light of the persistent challenge of diagnostic errors in primary care, the potential of AI-driven symptom checkers warrants further exploration. Drawing parallels from the secondary care sector, where structured interventions have reduced errors, AI symptom checkers could similarly offer GPs a systematic approach to consider differential diagnoses. Envisioning dual utilization, patients could benefit from an initial structured symptom assessment, while GPs might leverage these insights alongside their clinical acumen for a reinforced diagnostic process. However, the efficacy of this approach hinges on the tool's design, accuracy, and alignment with GP workflows, emphasizing the need for further research and pilot implementation.

Another study compared the performance of different symptom checkers with physicians’ appraisals and showed that GPs performed consistently better than the evaluated symptom checkers on diagnostic accuracy, while the triage advice (appropriateness and safety of urgency) of some apps was nearly comparable to that of the GPs [24]. Yet, most authors of this publication were affiliated to the company which produced the ‘best performing’ tool, thus, this result has to be considered cautiously. However, this study confirmed major differences between the tested symptom checkers as well [24].

Inconsistent ratings by the study participants also concerned the *helpfulness* of the symptom checker in medical visits. Patient-reported positive impacts on the consultation mainly concerned the pre-visit transmission of information to the GP, while the GPs considered this aspect less significant and gave more importance to the confirmation and support provided by the differential diagnoses. A GPs’ appreciation of digitally provided diagnostic information to reassure their own diagnosis was also confirmed in another study [25]. Interestingly, the GPs in our cohort perceived positive effects on the concerned patients in terms of a reinforcing aspect, and better preparation and attentiveness during the visit. This might allude to a promising potential in addition to the intention of strengthening patients’ self-management; the symptom checker could also be further developed to be used by patients in the GPs’ waiting room as a preparation for the medical visit by increasing self-reflection. Hence, a positive effect on patients could beneficially influence the consultation and the physician’s diagnostics.

It is pertinent to highlight the contrasting perceptions between doctors and patients concerning the value of *physical examination*. Patients often associate the tactile aspect of the examination with both diagnostic importance and affirmation of human connections. For many medical professionals, however, the patient's history predominantly informs their diagnosis, with physical examination acting mainly as a confirmatory step. This distinction might explain why GPs are more receptive to online consultations than patients, emphasizing the enduring importance of human touch and presence in the patient-doctor dynamic.

The *duration of the medical visit* was not reduced in most cases. The GPs even more frequently reported the visit to be prolonged than shortened; however, both were applied in relatively few cases, and most participants considered the duration to be adequate. A modest time-extending effect within the study setting is not surprising as it is an unusual approach that requires a certain degree of additional time and attention.

In general, it has not yet been clearly shown that the use of online triage tools can reduce the workload of GPs [15], and the required time has been mentioned by GPs as a barrier to the use of online diagnostic systems [25]. Nonetheless, future at-home use of the symptom checker (as intended) could meet these barriers if further development of the tool could reduce unnecessary face-to-face visits by supporting patients in self-management. Promising results were observed regarding the *usability* of the chatbot interface and a positive attitude towards the *future autonomous use of the symptom checker*. Although several patients indicated that they preferred face-to-face contact with the GP, some saw the benefits of at-home use, mainly regarding the potential to save time and medical visits. Controversial might be the aspect mentioned by the patients that an at-home chatbot result could be used by the GP as an aid in making a diagnosis. Although this might apply for some circumstances, it could also be impeding and require additional time and effort of the GPs if patients arrive in their office being fixed on a potentially incomplete or even erroneous pre-established diagnosis [25].

Similar to studies investigating the impact of increased virtual consultations during the COVID-19 pandemic on quality of care [4, 8], the GPs in our cohort frequently mentioned a concern regarding the indispensability of the physical examination and a preference for direct contact with the patient. Moreover, although the patients mainly expressed positive experiences regarding the intuitive and rapid use of the symptom checker, the GPs more often observed technical and procedural difficulties among patients, and an inability to correctly enter the symptoms. The GPs were, therefore, more reluctant towards recommend the symptom checker before or instead of a medical visit: only one out of ten GPs would probably suggest the use of the chatbot in case of doubt or suspicion of rare conditions.

Most GPs did not observe the potential of symptom checkers to *reduce unnecessary medical visits* in the future. This somewhat contradicts the perception of several patients who mentioned a potential in this regard (Supplementary Table I a), which was also confirmed by previous studies [15, 16, 25].

As other studies have shown and one GP mentioned in our cohort, physicians would appreciate the integration of symptom checkers and electronic decision support systems into their electronic health records to save time [25] and improve documentation [26]. However, it has to be taken into account that electronic decision support tools have also shown limited benefits for primary care [27–29].

From our analyses, no consistent results emerged regarding the suitability of the investigated symptom checker for *specific groups of patients*. As previously confirmed [30], a tendency was noted of male patients being more likely to use the symptom checker at home. Surprisingly, unlike previous research [16], older patients showed a more positive attitude towards the symptom checker, especially regarding its impact on medical visits. Yet, as a British population survey showed, older patients in primary care tend to be generally more satisfied than younger patients because, though they have higher expectations, they are also more likely to believe that these expectations are met [31]. This phenomenon of generally higher satisfaction among older patients might also have played a role in our study. However, the patients in our cohort mostly represented younger age groups (median age 47 years), which limits the validity of the conclusions regarding the utility of the applied chatbot for older people.

The GPs, who together with their medical assistants, perceived the entirety of the various patients’ difficulties in using the symptom checker, strongly agreed to recommend the symptom checker mostly for younger patients and those with skills in using digital devices. Nevertheless, previous studies have reported increasing use of digital health applications among older people [14]. Thus, adapting digital solutions by overcoming barriers for seniors may facilitate their use and should be fostered, as digital health technologies have shown beneficial effects on this age group, for example, enhanced communication, improved health management, and the promotion of independence [14].

### Strengths and limitations

This study was conducted in a real-life setting of primary care. This has to be considered a strength because, to date, few studies have assessed the implementation of symptom checkers in daily routine [15]. Moreover, the perspectives of both patients and GPs are outlined.

The GPs were offered the possibility of critically reflecting on their medical valuation. In addition, patients were given the chance to improve their self-perception and self-reflection by describing their symptoms and answering written health-related questions.

The most important limitations are the small sample size and the selection of participating GPs by convenience sampling. The number of participating patients was less than a quarter of the aspired sample size, which was mainly related to the external circumstances, as the timeline of the study according to the funding program placed the practice phase in early autumn 2021, when the pandemic-related workload also increased, impeding effective and motivating implementation. Despite an extension of the practice phase by two weeks the number of participating patients could not be substantially increased. However, compared to similar pilot studies, our sample size was still relatively large [27].

Another limitation pertains to the age distribution of the sample. While we highlighted the issue of an increasingly older population in the introduction, the age profile of our participants leaned towards a younger demographic. Specifically, a significant proportion of our participants were below 50 years of age. While we compared outcomes between younger and older age groups, defining those aged over 50 as “older” may not capture the nuances and challenges experienced by the older cohorts, typically categorized as over 65 or 75. This categorization was based on preliminary observations of technology acceptance and the onset of certain health risks. However, it is important to note that our findings might not be generalizable to the population aged 65 years and above. Future studies should include a more diverse age range, especially focusing on the elderly population, to ascertain the broader applicability of our findings.

As no screening logbook was kept for eligible patients, selection bias could not be further characterized. Patients who appeared unsuitable for participation and were, therefore, not even attempted to be recruited, were not included. Therefore, the findings are limited to a selected subgroup of primary care patients with acute symptoms who were able to handle a tablet personal computer.

This study focused on a single symptom checker and did not include comparisons between different tools.

Most of the participating GPs were working in a group office, although the experiences could have differed for GPs in single-handed practices.

Furthermore, we tested the chatbot’s utility in GP offices, which was intended to be autonomously used by patients before or independently from a medical visit. However, previous studies addressing symptom checkers are mainly placed in an experimental setting; therefore, this approach offers the advantages of embedding the use of a symptom checker within a real-life setting and of an appraisal by physicians, thus allowing for observations of potentially improved self-perception and self-reflection of the patients.

The patients were asked to provide their opinions after face-to-face contact with the GP (not immediately after the use of the symptom checker), as we aimed to investigate the perceived impacts of chatbot use on the perceived quality of the medical visit. However, the influence effects on patients cannot be excluded using this approach.

User satisfaction was measured using survey results, that is, by subjective ratings. In the post-visit evaluations, the option ‘neutral’ was the most frequently indicated answer for many items. Though this result per se provides a certain significance (neither clearly positive nor negative results), for future studies the use of a 4-point answer scale like in other studies [32] could be considered to obtain more meaningful results, e.g. ‘very satisfied,’ ‘rather satisfied,’ ‘rather dissatisfied, ‘ ‘very dissatisfied.’

This study offered the opportunity for digital anamnestic screening for patients that could be affected by COVID-19. However, as it was beyond the scope of the present feasibility study, we did not compare the number of patients with anamnestic risk for COVID-19 with the number of confirmed SARS-CoV-2 infections, and thus estimated the COVID-19-related accuracy of the symptom checker.

## Conclusion

The use of online symptom checkers is increasing and leveraged in various health systems with the aim of empowering patient self-management and relieving the burden on healthcare services. From our study, although several main pandemic-related barriers were met, which hampered its effective alignment with the workflow of GPs, the following conclusions were drawn:

- The use of a symptom checker was positively perceived by a small majority of the patients, mostly because of its rapid and intelligible use. A notable portion of patients would consider autonomous at-home use. However, the usefulness of the symptom checker for medical consultation was rated lower by patients than its user-friendliness and general satisfaction. On the other hand, the GPs observed positive preparative effects on patients.
- The GPs perceived points of criticism regarding the usefulness of patients’ self-management and reduction of unnecessary visits, mainly in terms of technical difficulties (which they reported more often than the patients themselves), and emphasized the indispensability of the physical examination for making a diagnosis in the majority of the presenting cases.
- The provided differential diagnoses were rated as helpful and adequate by the GPs in several cases, and were concordant with the evaluation of GPs in half of the cases.

Thus, the use of a symptom checker showed the potential to be further developed for the primary care setting, mainly regarding further simplified usability for all patient groups and appropriate differential diagnoses. Further studies should explore (i) whether the use of the chatbot can provide patients with a safe and beneficial option of self-management for the first appraisal of symptoms and (ii) whether and to what extent this might reduce the burden of unnecessary visits for GPs.

Moreover, our results underpin the previously stated requirement of clinical guidance in the development of symptom checkers to define preconditions for their widespread use and to guide policymakers in decisions concerning a larger-scale endorsement.

### Supplementary Information

**Additional file 1:** **Supplementary Tab. I.** Patients’ and GPs’ experiences with the symptom checker: free-text indications.

**Additional file 2. **Subgroup analyses according to patients' gender, age, and level of education.

**Additional file 3.** Questionnaire for patients.

**Additional file 4.** Post-visit questionnaire for general practitioners.

**Additional file 5.** Final questionnaire for general practitioners.

## Data Availability

The datasets used in the current study are available from the corresponding author upon reasonable request.

## Abbreviations

AI: Artificial intelligence
DDs: Differential diagnoses
eDS: Electronic decision support
EHR: Electronic health record
GP: General practitioner
IGP: Institute for General Practice and Public Health
IQR: Interquartile range

## References

[1] Napier J, Clinch M (2019). Job strain and retirement decisions in UK general practice. Occup Med (Lond).

[2] Jia H, Yu X, Jiang H, Yu J, Cao P, Gao S (2022). Analysis of factors affecting medical personnel seeking employment at primary health care institutions: developing human resources for primary health care. Int J Equity Health.

[3] Kontopantelis E, Roland M, Reeves D (2010). Patient experience of access to primary care: identification of predictors in a national patient survey. BMC Fam Pract.

[4] Chada BV (2017). Virtual consultations in general practice: embracing innovation, carefully. Br J Gen Pract.

[5] Pearl R (2014). Kaiser Permanente Northern California: current experiences with internet, mobile, and video technologies. Health Aff (Millwood).

[6] Atherton H, Brant H, Ziebland S, Bikker A, Campbell J, Gibson A, et al. In: The potential of alternatives to face-to-face consultation in general practice, and the impact on different patient groups: a mixed-methods case study. edn. Southampton (UK). 2018. 29889485

[7] Joy M, McGagh D, Jones N, Liyanage H, Sherlock J, Parimalanathan V (2020). Reorganisation of primary care for older adults during COVID-19: a cross-sectional database study in the UK. Br J Gen Pract.

[8] Tielker JM, Weber JP, Simon ST, Bausewein C, Stiel S, Schneider N (2021). Experiences, challenges and perspectives for ensuring end-of-life patient care: a national online survey with general practitioners in Germany. PLoS ONE.

[9] Moynihan R, Johansson M, Maybee A, Lang E, Legare F (2020). Covid-19: an opportunity to reduce unnecessary healthcare. BMJ.

[10] Mann DM, Chen J, Chunara R, Testa PA, Nov O (2020). COVID-19 transforms health care through telemedicine: Evidence from the field. J Am Med Inform Assoc.

[11] You Y, Gui X (2020). Self-diagnosis through ai-enabled chatbot-based symptom checkers: user experiences and design considerations. AMIA Annu Symp Proc.

[12] Munsch N, Martin A, Gruarin S, Nateqi J, Abdarahmane I, Weingartner-Ortner R (2020). Diagnostic accuracy of web-based COVID-19 symptom checkers: comparison study. J Med Internet Res.

[13] Semigran HL, Linder JA, Gidengil C, Mehrotra A (2015). Evaluation of symptom checkers for self diagnosis and triage: audit study. BMJ.

[14] Perlman A, Vodonos Zilberg A, Bak P, Dreyfuss M, Leventer-Roberts M, Vurembrand Y (2020). Characteristics and symptoms of app users seeking COVID-19-related digital health information and remote services: retrospective cohort study. J Med Internet Res.

[15] Gottliebsen K, Petersson G (2020). Limited evidence of benefits of patient operated intelligent primary care triage tools: findings of a literature review. BMJ Health Care Inform.

[16] Chambers D, Cantrell AJ, Johnson M, Preston L, Baxter SK, Booth A (2019). Digital and online symptom checkers and health assessment/triage services for urgent health problems: systematic review. BMJ Open.

[17] Mahlknecht A, Barbieri V, Engl A, Piccoliori G, Wiedermann CJ (2022). Challenges and experiences of general practitioners during the course of the Covid-19 pandemic: a northern Italian observational study-cross-sectional analysis and comparison of a two-time survey in primary care. Fam Pract.

[18] Hauser-Ulrich S, Kunzli H, Meier-Peterhans D, Kowatsch T (2020). A smartphone-based health care chatbot to promote self-management of chronic pain (SELMA): pilot randomized controlled trial. JMIR Mhealth Uhealth.

[19] Windak A, Frese T, Hummers E, Klemenc Ketis Z, Tsukagoshi S, Vilaseca J (2020). Academic general practice/family medicine in times of COVID-19 - Perspective of WONCA Europe. Eur J Gen Pract.

[20] Schmieding ML, Kopka M, Schmidt K, Schulz-Niethammer S, Balzer F, Feufel MA (2022). Triage accuracy of symptom checker apps: 5-year follow-up evaluation. J Med Internet Res.

[21] Hill MG, Sim M, Mills B (2020). The quality of diagnosis and triage advice provided by free online symptom checkers and apps in Australia. Med J Aust.

[22] Wallace W, Chan C, Chidambaram S, Hanna L, Iqbal FM, Acharya A (2022). The diagnostic and triage accuracy of digital and online symptom checker tools: a systematic review. NPJ Digit Med.

[23] Fraser H, Coiera E, Wong D (2018). Safety of patient-facing digital symptom checkers. Lancet.

[24] Gilbert S, Mehl A, Baluch A, Cawley C, Challiner J, Fraser H (2020). How accurate are digital symptom assessment apps for suggesting conditions and urgency advice? A clinical vignettes comparison to GPs. BMJ Open.

[25] McParland CR, Cooper MA, Johnston B (2019). Differential diagnosis decision support systems in primary and out-of-hours care: a qualitative analysis of the needs of key stakeholders in Scotland. J Prim Care Community Health.

[26] Kostopoulou O, Tracey C, Delaney BC (2021). Can decision support combat incompleteness and bias in routine primary care data?. J Am Med Inform Assoc.

[27] Henderson EJ, Rubin GP (2013). The utility of an online diagnostic decision support system (Isabel) in general practice: a process evaluation. JRSM Short Rep.

[28] Rieckert A, Reeves D, Altiner A, Drewelow E, Esmail A, Flamm M (2020). Use of an electronic decision support tool to reduce polypharmacy in elderly people with chronic diseases: cluster randomised controlled trial. BMJ.

[29] Rubin G, Walter FM, Emery J, Hamilton W, Hoare Z, Howse J (2021). Electronic clinical decision support tool for assessing stomach symptoms in primary care (ECASS): a feasibility study. BMJ Open.

[30] Ausserhofer D, Wiedermann W, Becker U, Vogele A, Piccoliori G, Wiedermann CJ (2022). Health information-seeking behavior associated with linguistic group membership: latent class analysis of a population-based cross-sectional survey in Italy, August to September 2014. Arch Public Health.

[31] Bowling A, Rowe G, McKee M (2013). Patients' experiences of their healthcare in relation to their expectations and satisfaction: a population survey. J R Soc Med.

[32] Miller S, Gilbert S, Virani V, Wicks P (2020). Patients' utilization and perception of an artificial intelligence-based symptom assessment and advice technology in a British primary care waiting room: exploratory pilot study. JMIR Hum Factors.

