# Supplementary material for: Supporting primary care through symptom checking artificial intelligence: a study of patient and physician attitudes in Italian general practice
Source: BMC Prim Care. 2023 Sep 4;24:174. doi: 10.1186/s12875-023-02143-0 (PMC10476397; doi:10.1186/s12875-023-02143-0)
Supplement: Supplementary file 1 — Additional file 1: Supplementary Tab. I. Patients’ and GPs’ experiences with the symptom checker: free-text indications. [file 12875_2023_2143_MOESM1_ESM.docx]

### Supplementary Tab. I: Patients’ and GPs’ experiences with the symptom checker: free-text indications

#### Supplementary Tab. I a: Free-text indications of patients and GPs (post-visit evaluation)

| General satisfaction with the symptom checker | | | | |
| --- | --- | --- | --- | --- |
|  | **Patients (n = 110)** | | **GPs (n = 122)** | |
| **Why satisfied** | - Easy use, intelligible, rapid - Precise, thorough questions - May help to save time - Interesting, encourages reflection - Well-functioning - Wider use in future, perhaps also at home - Feeling of protection - Conveyance of information [to the GP] | 14 (12.7%) ^i^  6 (5.5%)  3 (2.7%)  3 (2.7%)  2 (1.8%)  1 (0.9%)  1 (0.9%)  1 (0.9%) | - Adequate differential diagnoses / congruent with suspected diagnosis - Helpful, valid indications - Interesting DDs - Motivates [the patients] to reflect on health - Easy use of the symptom checker | 27 (22.1%) ^i^  4 (3.3%)  3 (2.5%)  1 (0.8%)  1 (0.8%) |
| **Why dissatisfied** | - Impersonal, cannot replace personal contact with GP - Questions too generic - Complex - Paper preferred - Inadequate questions | 3 (2.7%) ^i^  2 (1.8%)  2 (1.8%)  1 (0.9%)  1 (0.9%) | - The symptom checker did not provide adequate DDs - The patient was not able to enter symptoms adequately - Too unspecific - Inadequate questions - Technical issues - Impracticable | 13 (10.7%) ^i^  8 (6.6%)  5 (4.1%)  2 (1.6%)  1 (0.8%)  1 (0.8%) |
| Impact of the chatbot use on the quality of the medical visit | | | | |
|  | **Patients (n = 111)** | | **GPs (n = 122)** | |
| **Why positively** | - Pre-visit information for the GP - Time saved - Aid to the GP for improved care - Aid to the GP for evaluating the patient's health status - Feeling of taken seriously - Interest in innovation of the medical crew | 8 (7.2%)  3 (2.7%)  2 (1.8%)  1 (0.9%)  1 (0.9%)  1 (0.9%) | - Help / reassurance by DDs - Positive effects on patients: preparation, attentiveness, reassurance - Pre-visit information - Easy use of the symptom checker - Patient appreciated the use of an IT application - Exclusion of COVID-19 | 13 (10.7%)  9 (7.4%)  1 (0.8%)  1 (0.8%)  1 (0.8%)  1 (0.8%) |
| **Why negatively** | - | - | - Focus on an inadequate problem - Distraction from the real problem - Time delay | 1 (0.8%)  1 (0.8%)  1 (0.8%) |
| Helpfulness of the symptom checker for the medical visit | | | | |
|  | **Patients (n = 110)** | | **GPs (n = 122)** | |
| **Why helpful:** | - Comprehensive pre-information for the GP - Exclusion of COVID-19 infection | 7 (6.4%)  1 (0.9%) | - Confirmation of suspected diagnosis - Helpful list of DDs, decision support - DDs rapidly obtained - COVID-19 risk assessment | 10 (8.2%)  9 (7.4%)  3 (2.5%)  1 (0.8%) |
| **Why not helpful:** | - Questions were not related to current problem/focused on COVID-19 - No additional benefit - Personal contact [with the GP] preferred - No diagnosis received / not helpful for diagnosis | 5 (4.5%)  3 (2.7%)  3 (2.7%)  3 (2.7%) | - DD inadequate - Inappropriate indications by the patient - No recommendations provided - No information added - Anamnesis insufficient | 13 (10.7%)  3 (2.5%)  3 (2.5%)  2 (1.6%)  1 (0.8%) |
| Disturbance of the medical visit by the use of the symptom checker | | | | |
|  | **Patients (n = 110)** | | **GPs (n = 122)** | |
| **Why disturbing:** | - Questions [of the chatbot] could be asked personally | 1 (0.9%) | - The information generated by the chatbot was inappropriate | 1 (0.8%) |
| Future at-home use of the symptom checker as an aid to the appraisal of health problems | | | | |
|  | **Patients (n = 115)** | | **-** | |
| **Why yes:** | - To save time, e.g. waiting time in the GP office - To reduce medical visits - As an aid to the GP - May be helpful (without further specification) - For a first appraisal - For after-treatment - Good concept which needs further development | 8 (7.0%)  8 (7.0%)  8 (7.0%)  4 (2.8%)  2 (1.7%)  1 (0.7%)  1 (0.7%) | - | |
| **Why not:** | - Personal contact preferred - No additional benefit - Too complicated - I use [chatbots] for other purposes - Not for a first contact / new health problem - Not sufficient confidence | 14 (12.2%)  4 (3.5%)  2 (1.7%)  1 (0.9%)  1 (0.9%)  1 (0.9%) | - | |
| Other experiences | | | | |
| Patients (n = 116) | | | GPs (n = 122) | |
| - User-friendly, rapid - New experience - Most of the questions aimed at identifying COVID-19 - Too inaccurate, medical visit is better - The questions should focus on the concerned part of the body - It is no option for patients | | 1 (0.9%)  1 (0.9%)  1 (0.9%)  1 (0.9%)  1 (0.9%)  1 (0.9%) | - The symptom checker was not helpful for the visit - The patient has not entered any symptoms - Useful, no problems - Use of the chatbot and entry of symptoms was difficult for patients - Inadequate questions of the symptom checker - No symptom report due to estimated COVID-19 risk - No list of symptoms was generated | 9 (7.4%)  9 (7.4%)  7 (5.7%)  5 (4.1%)  3 (2.5%)  2 (1.6%)  2 (1.6%) |

*GPs* General practitioners, *DDs* Differential diagnoses

^i^ The reported percentages of the free-text indications are related to the number of patients respectively GPs (n) who answered to the corresponding numeric item of the questionnaire.

#### Supplementary Tab. I b: Free-text indications of GPs (final evaluation)

| GPs’ general satisfaction with the symptom checker (n=10) | | |
| --- | --- | --- |
| **Why satisfied:** | - Helpful for differential diagnosis - Confirmation of GPs' way of working | 2 (20.0%)  1 (10.0%) |
| **Why dissatisfied:** | - Many patients fulfilled the exclusion criteria - Time problems due to extensive workload - Logistically inconvenient for GPs/assistants - Logistically inconvenient for patients - Technical problems during the practice phase - Questions and differential diagnoses not helpful - Only helpful for determinate patients / age groups | 4 (40.0%) ^i^  4 (40.0%)  4 (40.0%)  3 (30.0%)  3 (30.0%)  3 (30.0%)  1 (10.0%) |
| Do GPs consider the use of the symptom checker as helpful for patients’ self-management (n=10) | | |
| **Why helpful:** | - Useful if it helps to differentiate and to recognise danger - Useful if integrated in the EHR with implemented eDS + guidelines and linked to the results of diagnostic exams | 2 (20.0%)  1 (10.0%) |
| **Why not helpful:** | - Too complicated, technically not feasible - Correct diagnosis not feasible without clinical examination - Direct contact more appreciated - Not useful during the visit because patients wanted to know all chatbot diagnoses, also the not correct ones | 4 (40%)  2 (20%)  2 (20%)  2 (20%) |
| Probability that GPs recommend the use of the chatbot before or as alternative to a medical visit (n=10) | | |
| **Why yes:** | - Useful in case of doubt or rare conditions - Only if results are approved by the doctor | 1 (10.0%)  1 (10.0%) |
| **Why not:** | - Correct diagnosis and clinical appraisal not feasible without clinical examination - The written explanation of their symptoms is difficult for the patients - Not suitable for anxious and insecure patients - Patients are not able to do it by themselves, many needed medical assistant - The 'gut instinct' prefers the medical visit | 1 (10.0%)  1 (10.0%)  1 (10.0%)  1 (10.0%)  1 (10.0%) |
| Do GPs consider specific patient groups as especially suited for the use of the symptom checker (n=10) | | |
| **If yes, which patients:** | - Young patients with expertise in using digital devices - Patients with benign symptoms/conditions - Patients with complex symptoms prior to specialist visit - Patients with rheumatic diseases - Patients with respiratory infections, arthralgia, back pain, light headache | 5 (50.0%)  2 (20.0%)  1 (10.0%)  1 (10.0%)  1 (10.0%) |
| GPs’ general experiences with the symptom checker (n=10) | | |
| - Difficulties of implementing the study during the pandemic due to the high workload - Interesting method - Too frequent exclusion of patients (exclusion criteria, COVID-19 medium/high risk) - Time investment and presentation of the reports was good - Results were unsatisfactory for the most common problems in general practice (missing time aspect) - Clinical examination is indispensable - Impossibility of entry correction in the symptom checker | | 4 (40.0%)  3 (30.0%)  2 (20.0%)  1 (10.0%)  1 (10.0%)  1 (10.0%)  1 (10.0%) |

*GPs* General practitioners, *EHR* Electronic health record, *eDS* Electronic decision support

^i^ The reported percentages of the free-text indications are related to the number of GPs (n) who answered to the corresponding numeric item of the questionnaire.
