# Supplementary material for: Supporting primary care through symptom checking artificial intelligence: a study of patient and physician attitudes in Italian general practice
Source: BMC Prim Care. 2023 Sep 4;24:174. doi: 10.1186/s12875-023-02143-0 (PMC10476397; doi:10.1186/s12875-023-02143-0)
Supplement: Supplementary file 2 — Additional file 2. Subgroup analyses according to patients' gender, age, and level of education. [file 12875_2023_2143_MOESM2_ESM.docx]

### **Supplementary Tab. II: Subgroup analyses**

#### Supplementary Tab. II a: Subgroup analysis according to patients’ gender

| **Have patients already used a symptom checker in the past** | **Male** | **Female** | p-value |
| --- | --- | --- | --- |
| Yes, several times | 5 (9.6%) | 4 (6.3%) | 0.625 ^i^ |
| Yes, once | 3 (5.8%) | 2 (3.2%) |  |
| No, never | 44 (84.6%) | 57 (90.5%) |  |
| *n total* | *52* | *63* |  |
| **Patients’ general satisfaction with the symptom checker** | **Male** | **Female** | **p-value** |
| Very satisfied | 11 (22.4%) | 10 (16.4%) | 0.820 ^i^ |
| Rather satisfied | 13 (26.5%) | 20 (32.8%) |  |
| Neutral | 22 (44.9%) | 29 (47.5%) |  |
| Rather dissatisfied | 2 (4.1%) | 1 (1.6%) |  |
| Very dissatisfied | 1 (2.0%) | 1 (1.6%) |  |
| *n total* | *49* | *61* |  |
| **Impact of the chatbot use on the quality of the medical visit** | **Male** | **Female** | **p-value** |
| Very positive | 3 (5.9%) | 3 (5.0%) | 0.819 ^i^ |
| Rather positive | 11 (21.6%) | 12 (20.0%) |  |
| Neutral | 37 (72.5%) | 44 (73.3%) |  |
| Rather negative | 0 (0.0%) | 1 (1.7%) |  |
| Very negative | 0 (0.0%) | 0 (0.0%) |  |
| *n total* | *51* | *60* |  |
| Was the **symptom checker** helpful for the medical visit | **Male** | **Female** | **p-value** |
| Very helpful | 3 (6.1%) | 2 (3.3%) | 0.537 ^i^ |
| Rather helpful | 8 (16.3%) | 15 (24.6%) |  |
| Neutral | 30 (61.2%) | 32 (52.5%) |  |
| Rather not helpful | 2 (4.1%) | 6 (9.8%) |  |
| Not helpful at all | 6 (12.2%) | 6 (9.8%) |  |
| *n total* | *49* | *61* |  |
| Was the **symptom checker** disturbing for the medical visit | **Male** | **Female** | **p-value** |
| Very disturbing | 0 (0.0%) | 0 (0.0%) | 0.286 ^i^ |
| Rather disturbing | 0 (0.0%) | 3 (5.0%) |  |
| Neutral | 17 (34.0%) | 16 (26.7%) |  |
| Rather not disturbing | 6 (12.0%) | 11 (18.3%) |  |
| Not disturbing at all | 27 (54.0%) | 30 (50.0%) |  |
| *n total* | *50* | *60* |  |
| At-home use of the chatbot for appraisal of health problems | **Male** | **Female** | **p-value** |
| Yes, surely | 16 (30.8%) | 12 (19.0%) | 0.247 ^i^ |
| Rather yes | 13 (25.0%) | 13 (20.6%) |  |
| Neutral | 13 (25.0%) | 14 (22.2%) |  |
| Rather not | 8 (15.4%) | 20 (31.7%) |  |
| Absolutely not | 2 (3.8%) | 4 (6.3%) |  |
| *n total* | *52* | *63* |  |

^i^ Chi square test

#### Supplementary Tab. II b: Subgroup analysis according to patients’ age (dichotomised age groups and correlation)

| **Have patients already used a symptom checker in the past** | **≤ 50 years** | **> 50 years** | p-value |
| --- | --- | --- | --- |
| Yes, several times | 7 (10.1%) | 2 (4.6%) | 0.370 ^i^ |
| Yes, once | 2 (2.9%) | 3 (7.0%) |  |
| No, never | 60 (87.0%) | 38 (88.4%) |  |
| *n total* | *69* | *43* |  |
| **Patients’ general satisfaction with the symptom checker** | **≤ 50 years** | **> 50 years** | p-value |
| Very satisfied | 11 (16.2%) | 10 (25.6%) | 0.442 ^i^ |
| Rather satisfied | 18 (26.5%) | 12 (30.8%) |  |
| Neutral | 35 (51.5%) | 16 (41.0%) |  |
| Rather dissatisfied | 3 (4.4%) | 0 (0.0%) |  |
| Very dissatisfied | 1 (1.5%) | 1 (2.6%) |  |
| *n total* | *68* | *39* |  |
| **Impact of the chatbot use on the quality of the medical visit** | **≤ 50 years** | **> 50 years** | p-value |
| Very positive | 4 (5.9%) | 2 (5.0%) | 0.342 ^i^ |
| Rather positive | 11 (16.2%) | 12 (30.0%) |  |
| Neutral | 52 (76.5%) | 26 (65.0%) |  |
| Rather negative | 1 (1.5%) | 0 (0.0%) |  |
| Very negative | 0 (0.0%) | 0 (0.0%) |  |
| *n total* | *68* | *40* |  |
| Was the **symptom checker** helpful for the medical visit | **≤ 50 years** | **> 50 years** | p-value |
| Very helpful | 2 (3.0%) | 3 (7.3%) | 0.735 ^i^ |
| Rather helpful | 13 (19.7%) | 10 (24.4%) |  |
| Neutral | 37 (56.1%) | 22 (53.7%) |  |
| Rather not helpful | 6 (9.1%) | 2 (4.9%) |  |
| Not helpful at all | 8 (12.1%) | 4 (9.8%) |  |
| *n total* | *66* | *41* |  |
| Was the **symptom checker** disturbing for the medical visit | **≤ 50 years** | **> 50 years** | p-value |
| Very disturbing | 0 (0.0%) | 0 (0.0%) | 0.216 ^i^ |
| Rather disturbing | 2 (3.0%) | 1 (2.4%) |  |
| Neutral | 16 (24.3%) | 15 (36.6%) |  |
| Rather not disturbing | 14 (21.1%) | 3 (7.3%) |  |
| Not disturbing at all | 34 (51.5%) | 22 (53.7%) |  |
| *n total* | *66* | *41* |  |
| At-home use of the **chatbot** for appraisal of health problems | **≤ 50 years** | **> 50 years** | p-value |
| Yes, surely | 17 (24.6%) | 11 (25.6%) | 0.814 ^i^ |
| Rather yes | 13 (18.8%) | 12 (27.9%) |  |
| Neutral | 16 (23.2%) | 9 (20.9%) |  |
| Rather not | 19 (27.5%) | 9 (20.9%) |  |
| Absolutely not | 4 (5.8%) | 2 (4.6%) |  |
| *n total* | *69* | *43* |  |
| **CORRELATION with patients’ age** | **Spearman’s Rho** | | p-value |
| Have patients already used a symptom checker in the past | - 0.022 | | 0.821 ^ii^ |
| Patients’ general satisfaction with the symptom checker | 0.104 | | 0.287 ^ii^ |
| Impact of the chatbot use on the quality of the medical visit | 0.148 | | 0.127 ^ii^ |
| Was the symptom checker helpful for the medical visit | 0.096 | | 0.327 ^ii^ |
| Was the symptom checker disturbing for the medical visit | 0.010 | | 0.918 ^ii^ |
| At-home use of the chatbot for appraisal of health problems | - 0.009 | | 0.925 ^ii^ |

^i^ Chi square test, ^ii^ Spearman correlation

#### Supplementary Tab. II c: Subgroup analysis according to patients’ level of education

| **Have patients already used a symptom checker in the past** | **Low – medium education level** | **High**  **education level** | p-value |
| --- | --- | --- | --- |
| Yes, several times | 2 (3.6%) | 7 (11.9%) | 0.242 ^i^ |
| Yes, once | 3 (5.5%) | 2 (3.4%) |  |
| No, never | 50 (90.9%) | 50 (84.7%) |  |
| *n total* | *55* | *59* |  |
| **Patients’ general satisfaction with the symptom checker** | **Low – medium education level** | **High**  **education level** | **p-value** |
| Very satisfied | 10 (19.2%) | 11 (19.3%) | 0.518 ^i^ |
| Rather satisfied | 17 (32.7%) | 15 (26.3%) |  |
| Neutral | 22 (42.3%) | 29 (50.9%) |  |
| Rather dissatisfied | 1 (1.9%) | 2 (3.5%) |  |
| Very dissatisfied | 2 (3.8%) | 0 (0.0%) |  |
| *n total* | *52* | *57* |  |
| **Impact of the chatbot use on the quality of the medical visit** | **Low – medium education level** | **High**  **education level** | **p-value** |
| Very positive | 3 (5.7%) | 3 (5.3%) | 0.188 ^i^ |
| Rather positive | 15 (28.3%) | 8 (14.0%) |  |
| Neutral | 34 (64.2%) | 46 (80.7%) |  |
| Rather negative | 1 (1.9%) | 0 (0.0%) |  |
| Very negative | 0 (0.0%) | 0 (0.0%) |  |
| *n total* | *53* | *57* |  |
| Was the **symptom checker** helpful for the medical visit | **Low – medium education level** | **High**  **education level** | **p-value** |
| Very helpful | 4 (7.5%) | 1 (1.8%) | 0.183 ^i^ |
| Rather helpful | 14 (26.4%) | 9 (16.1%) |  |
| Neutral | 28 (52.8%) | 33 (58.9%) |  |
| Rather not helpful | 4 (7.5%) | 4 (7.1%) |  |
| Not helpful at all | 3 (5.7%) | 9 (16.1%) |  |
| *n total* | *53* | *56* |  |
| Was the **symptom checker** disturbing for the medical visit | **Low – medium education level** | **High**  **education level** | **p-value** |
| Very disturbing | 0 (0.0%) | 0 (0.0%) | 0.243 ^i^ |
| Rather disturbing | 0 (0.0%) | 3 (5.5 %) |  |
| Neutral | 14 (25.9%) | 18 (32.7%) |  |
| Rather not disturbing | 10 (18.5%) | 7 (12.7%) |  |
| Not disturbing at all | 30 (55.6%) | 27 (49.1%) |  |
| *n total* | *54* | *55* |  |
| At-home use of the chatbot for appraisal of health problems | **Low – medium education level** | **High**  **education level** | **p-value** |
| Yes, surely | 14 (25.5%) | 14 (23.7%) | 0.968 ^i^ |
| Rather yes | 11 (20.0%) | 15 (25.4%) |  |
| Neutral | 14 (25.5%) | 13 (22.0%) |  |
| Rather not | 13 (23.6%) | 14 (23.7%) |  |
| Absolutely not | 3 (5.5%) | 3 (5.1%) |  |
| *n total* | *55* | *59* |  |

Low - medium education level: basic education, intermediate school, or vocational college

High education level: high school or university

^i^ Chi square test
