# Supplementary material for: Supporting primary care through symptom checking artificial intelligence: a study of patient and physician attitudes in Italian general practice
Source: BMC Prim Care. 2023 Sep 4;24:174. doi: 10.1186/s12875-023-02143-0 (PMC10476397; doi:10.1186/s12875-023-02143-0)
Supplement: Supplementary file 3 — Additional file 3. Questionnaire for patients. [file 12875_2023_2143_MOESM3_ESM.docx]

Study ‚Symptom Checking in General Practice’

**Questionnaire for patients**

## **Demographic information**

**1. Gender**

Male  Female

**2. Age in years**

______________

**3. Linguistic group (mother language)**

German  Italian  Ladin  Other language

**4. Nationality**

Italian  Other

**5. Educational level**

Basic education

Intermediate school

Vocational college

High school

University

**6. What is your current working situation?**

Employee

Freelancer

Maternal leave / housewife

Professional education

Student

Job seeking

Retired

## **Health-related information**

**7. Where do you primarily seek information in case of health problems?**

I ask my general practitioner (GP)

I ask another physician

Pharmacist

Family members or friends

Print media (books, journals)

Homepages of medical specialist societies

Google

YouTube

social media, chats

Others: ________________________________________________________________________

**8. How would you rate your health status in general?**

| Very poor | Poor | Mediocre | Good | Very good |
| --- | --- | --- | --- | --- |
|  |  |  |  |  |

**9. Do you suffer from any of the following chronic conditions?**

Pulmonal diseases

Cardiovascular diseases (e.g. coronary heart disease, atrial fibrillation, heart failure, cerebro-vascular disease, ictus cerebri)

Arterial hypertension

Renal diseases

Hepatic diseases

Immunologic diseases

Metabolic diseases (e.g. diabetes mellitus, adipositas)

Allergies

Oncologic diseases

Psychologic diseases, depression, or anxiety disorder

Others: ________________________________________________________________________

None of these

**10. What was the reason for seeking your GP today?**

__________________________________________________________________________________

__________________________________________________________________________________

**11. What did the GP recommend at the end of the today’s medical visit?**

Discharge at home in self-observation

Telephonic re-contact with the GP

Physical re-contact with the GP (medical visit)

Prescription of a medical therapy (oral drug, unguent, …)

Prescription of / referral to diagnostic measures (e.g. radiologic or laboratory exams)

Referral to a medical specialist

Referral to an emergency department

**11a. Level of urgency in case of referral to a medical specialist or to a diagnostic procedure**

Within 24 hours

Within 10 days

Within 30-60 days

Within 120 days

## **Questions relating to the experience with the digital health assistant (chatbot)**

**12. You have used a digital health assistant (chatbot) today before the medical visit. Have you already used a similar chatbot in the past?**

Yes, several times

Yes, once

No, never

**13. You have used a digital health assistant (chatbot) today before the medical visit. How satisfied were you with the chatbot in general?**

| Very dissatisfied | Rather dissatisfied | Neutral | Rather satisfied | Very satisfied |
| --- | --- | --- | --- | --- |
|  |  |  |  |  |

**13a. Why dissatisfied?**

__________________________________________________________________________________

__________________________________________________________________________________

**13b. Why satisfied?**

__________________________________________________________________________________

__________________________________________________________________________________

**14. In your opinion, how has the use of the chatbot influenced the quality of the medical visit?**

| Very negatively | Rather negatively | | Neutral | Rather positively | Very positively |
| --- | --- | --- | --- | --- | --- |
|  | |  |  |  |  |

**14a. Why negatively?**

__________________________________________________________________________________

__________________________________________________________________________________

**14b. Why positively?**

__________________________________________________________________________________

__________________________________________________________________________________

**15. Was the use of the chatbot helpful for the medical visit?**

| Not helpful at all | Rather not helpful | Neutral | Rather helpful | Very helpful |
| --- | --- | --- | --- | --- |
|  |  |  |  |  |

**15a. Why not helpful?**

__________________________________________________________________________________

__________________________________________________________________________________

**15b. Why helpful?**

__________________________________________________________________________________

__________________________________________________________________________________

**16. Was the use of the chatbot disturbing for the medical visit?**

| Not disturbing  at all | Rather not disturbing | Neutral | Rather  disturbing | Very  disturbing |
| --- | --- | --- | --- | --- |
|  |  |  |  |  |

**16a. Why disturbing?**

__________________________________________________________________________________

__________________________________________________________________________________

**17. Did you have the impression that your GP has considered the indications which you gave on the tablet before the medical visit?**

Yes  No

**18. How was the duration of the medical visit after the use of the chatbot, compared to the duration which you expected?**

| Much shorter | Rather shorter | Unvaried | Rather longer | Much longer |
| --- | --- | --- | --- | --- |
|  |  |  |  |  |

**18a. How would you value the duration of the today’s medical visit?**

Too short

Adequate

Too long

**19. In the future: would you use a chatbot also at home to get an aid in the appraisal of health problems?**

| Absolutely not | Rather not | Neutral | Rather yes | Yes, surely |
| --- | --- | --- | --- | --- |
|  |  |  |  |  |

**19a. Why not?**

__________________________________________________________________________________

__________________________________________________________________________________

**19b. Why yes?**

__________________________________________________________________________________

__________________________________________________________________________________

**20. How satisfied are you with the usability of the digital chatbot on the tablet?**

| Very dissatisfied | Rather dissatisfied | Neutral | Rather satisfied | Very satisfied |
| --- | --- | --- | --- | --- |
|  |  |  |  |  |

**21.** **Do you want to report other experiences with the chatbot?**

__________________________________________________________________________________

__________________________________________________________________________________

__________________________________________________________________________________
