# Supplementary material for: Supporting primary care through symptom checking artificial intelligence: a study of patient and physician attitudes in Italian general practice
Source: BMC Prim Care. 2023 Sep 4;24:174. doi: 10.1186/s12875-023-02143-0 (PMC10476397; doi:10.1186/s12875-023-02143-0)
Supplement: Supplementary file 4 — Additional file 4. Post-visit questionnaire for general practitioners. [file 12875_2023_2143_MOESM4_ESM.docx]

Study ‚Symptom Checking in General Practice’

**Questionnaire for general practitioners**

## **Questions relating to the patient**

**1. How would you rate the health status of the patient in general?**

| Very poor | Poor | Mediocre | Good | Very good |
| --- | --- | --- | --- | --- |
|  |  |  |  |  |

**2. What was the reason of the patient for seeking you today?**

__________________________________________________________________________________

__________________________________________________________________________________

## **Questions relating to the experience with the digital health assistant (chatbot)**

**3. Was the result of the chatbot concordant with your clinical appraisal?**

| Not concordant  at all | Rather not concordant | Rather  concordant | Completely concordant |
| --- | --- | --- | --- |
|  |  |  |  |

**4. How would you rate the quality of the chatbot result from a clinical point of view?**

| Very  inappropriate | Rather  inappropriate | Neutral | Rather  appropriate | Very  appropriate |
| --- | --- | --- | --- | --- |
|  |  |  |  |  |

**5. How satisfied are you with the chatbot result regarding this specific patient?**

| Very dissatisfied | Rather dissatisfied | Neutral | Rather satisfied | Very satisfied |
| --- | --- | --- | --- | --- |
|  |  |  |  |  |

**5a. Why dissatisfied?**

__________________________________________________________________________________

__________________________________________________________________________________

**5b. Why satisfied?**

__________________________________________________________________________________

__________________________________________________________________________________

**6. In your opinion, how has the use of the chatbot influenced the quality of the medical visit?**

| Very negatively | Rather negatively | | Neutral | Rather positively | Very positively |
| --- | --- | --- | --- | --- | --- |
|  | |  |  |  |  |

**6a. Why negatively?**

__________________________________________________________________________________

__________________________________________________________________________________

**6b. Why positively?**

__________________________________________________________________________________

__________________________________________________________________________________

**7. Was the information generated by the chatbot helpful for the medical visit?**

| Not helpful at all | Rather not helpful | Neutral | Rather helpful | Very helpful |
| --- | --- | --- | --- | --- |
|  |  |  |  |  |

**7a. Why not helpful?**

__________________________________________________________________________________

__________________________________________________________________________________

**7b. Why helpful?**

__________________________________________________________________________________

__________________________________________________________________________________

**8. Was the information generated by the chatbot disturbing for the medical visit?**

| Not disturbing  at all | Rather not disturbing | Neutral | Rather  disturbing | Very  disturbing |
| --- | --- | --- | --- | --- |
|  |  |  |  |  |

**8a. Why disturbing?**

__________________________________________________________________________________

__________________________________________________________________________________

**9. How was the duration of the visit after the use of the chatbot, compared to the duration which you expected?**

| Much shorter | Rather shorter | Unvaried | Rather longer | Much longer |
| --- | --- | --- | --- | --- |
|  |  |  |  |  |

**9a. How would you value the duration of the medical visit?**

Too short

Adequate

Too long

**10.** **Do you want to report other experiences with the chatbot?**

__________________________________________________________________________________

__________________________________________________________________________________

__________________________________________________________________________________
