# Supplementary material for: Supporting primary care through symptom checking artificial intelligence: a study of patient and physician attitudes in Italian general practice
Source: BMC Prim Care. 2023 Sep 4;24:174. doi: 10.1186/s12875-023-02143-0 (PMC10476397; doi:10.1186/s12875-023-02143-0)
Supplement: Supplementary file 5 — Additional file 5. Final questionnaire for general practitioners. [file 12875_2023_2143_MOESM5_ESM.docx]

Study ‚Symptom Checking in General Practice’

**Questionnaire for general practitioners**

## **Demographic information**

**1. Gender**

Male  Female

**2. Age in years**

______________

**3. Linguistic group (mother language)**

German  Italian  Ladin  Other language

**4. Number of assisted patients**

______________

**5. Location of the GP office**

Rural area  Urban area

**6. Practice organisation**

Group office  Network of GP offices  Single-handed office

**7. Duration of professional activity in years**

______________

## **Questions relating to the experience with the digital health assistant (chatbot)**

**8. How satisfied are you with the chatbot in general?**

| Very dissatisfied | Rather dissatisfied | Neutral | Rather satisfied | Very satisfied |
| --- | --- | --- | --- | --- |
|  |  |  |  |  |

**8a. Why dissatisfied?**

__________________________________________________________________________________

__________________________________________________________________________________

**8b. Why satisfied?**

__________________________________________________________________________________

__________________________________________________________________________________

**9. Do you consider the use of the chatbot as helpful for patients’ self-management?**

| Not helpful at all | Rather not helpful | | Neutral | Helpful | Very helpful |
| --- | --- | --- | --- | --- | --- |
|  | |  |  |  |  |

**9a. Why not helpful?**

__________________________________________________________________________________

__________________________________________________________________________________

**9b. Why helpful?**

__________________________________________________________________________________

__________________________________________________________________________________

**10. How probable is it that you recommend the use of the chatbot before or as alternative to a medical visit for appropriate non-urgent medical problems?**

| Not at all | Rather not | Neutral | Rather yes | Yes, surely |
| --- | --- | --- | --- | --- |
|  |  |  |  |  |

**10a. Why not?**

__________________________________________________________________________________

__________________________________________________________________________________

**10b. Why yes?**

__________________________________________________________________________________

__________________________________________________________________________________

**11. Do you consider the use of the chatbot helpful to reduce unnecessary medical visits in the future?**

| Not helpful at all | Rather not helpful | Neutral | Helpful | Very helpful |
| --- | --- | --- | --- | --- |
|  |  |  |  |  |

**12. Do you consider specific groups pf patients (e.g. age groups, determined conditions) as especially suited for the use of the chatbot?**

No  Yes

**12a. If yes, which groups of patients?**

__________________________________________________________________________________

__________________________________________________________________________________

**13.** **Do you want to report other experiences with the chatbot?**

__________________________________________________________________________________

__________________________________________________________________________________

__________________________________________________________________________________
